# Anti-cancer effects of *Bifidobacterium* species in colon cancer cells and a mouse model of carcinogenesis

**DOI:** 10.1371/journal.pone.0232930

**Published:** 2020-05-13

**Authors:** Asadollahi Parisa, Ghanavati Roya, Rohani Mahdi, Razavi Shabnam, Esghaei Maryam, Talebi Malihe

**Affiliations:** 1 Microbial Biotechnology Research Centre, Iran University of Medical Sciences, Tehran, Iran; 2 Department of Microbiology, School of Medicine, Iran University of Medical Sciences, Tehran, Iran; 3 Behbahan Faculty of Medical Science, Behbahan, Iran; 4 Department of Microbiology, Pasteur Institute of Iran, Tehran, Iran; 5 Department of Virology, School of Medicine, Iran University of Medical Sciences, Tehran, Iran; Duke University School of Medicine, UNITED STATES

## Abstract

**Introduction:**

Probiotics are suggested to prevent colorectal cancer (CRC). This study aimed to investigate the anticancer properties of some potential probiotics *in vitro* and *in vivo*.

**Materials and methods:**

Anticancer effects of potential probiotic groups were investigated following of in LS174T cancer cells compared to IEC-18 normal cells. 1. a single strain of *Bifidobacterium*. *breve*, 2. a single strain of *Lactobacillus*. *reuteri*, 3. a cocktail of 5 strains of Lactobacilli (LC), 4. a cocktail of 5 strains of Bifidobacteria (BC), 5. a cocktail of 10 strains from *Lactobacillus* and *Bifidobacterium* (L+B). Apoptosis rate, EGFR, HER-2 and *PTGS-2* (COX-2 protein) expression levels were assessed as metrics of evaluating anticancer properties. Effect of BC, as the most effective group *in vitro*, was further assessed in mice models.

**Results:**

BC induced ~21% and only ~3% apoptosis among LS174T and IEC-18 cells respectively. BC decreased the expression of *EGFR* by 4.4 folds, *HER-2* by 6.7 folds, and *PTGS-2* by 20 folds among the LS174T cells. In all these cases, BC did not interfere significantly with the expression of the genes in IEC-18 cells. This cocktail has caused only 1.1 folds decrease, 1.8 folds increase and 1.7 folds decrease in *EGFR*, *HER-2* and *PTGS-2* expression, respectively. Western blot analysis confirmed these results in the protein level. BC significantly ameliorated the disease activity index, restored colon length, inhibited the increase in incidence and progress of tumors to higher stages and grades.

**Conclusions:**

BC was the most efficient treatment in this study. It had considerable “protective” anti-cancer properties and concomitantly down regulated *EGFR*, *HER-2* and *PTGS-2* (COX-2), while having significant anti-CRC effects on CRC mice models. In general, this potential probiotic could be considered as a suitable nutritional supplement to treat and prevent CRC.

## Introduction

Colorectal cancer (CRC) is the third most common type of cancer, being surpassed by only lung and breast cancers, and the second cause of cancer-related deaths worldwide [1]. There are abundant data regarding the association of CRC with dysbiosis of the gut microbiota [1, 2]. Probiotic bacteria are defined as “live microorganisms that when consumed in sufficient amounts exert health benefits to the host”, and most commonly belong to the lactic acid bacteria (LAB), including *Lactobacillus* and *Bifidobacterium* spp. Evidence from many studies suggest a preventive role for LAB probiotics in the onset of CRC both *in vitro* and *in vivo* [3–8]. Some of the suggested mechanisms probiotics exert their beneficial effects on CRC prevention include improvement of the host’s immune response, induction of apoptosis, and inhibition of tyrosine kinase signaling pathways [1, 8, 9]. One of these important CRC- involved signaling pathways, suggested to be inhibited by some probiotics, is the epidermal growth factor receptor (EGFR) pathway. The EGF receptor family has four consisting members: EGFR/ErbB1, HER1, HER2/ ErbB2/Neu, HER-3/ErbB3 and HER-4/ErbB4. All of these receptors contain an extracellular ligand-binding region, a single membrane-spanning region, and a cytoplasmic tyrosine kinase-containing domain [10]. Briefly, ligand binding induces dimerization of ErbB receptors, either as homo- (e.g. two EGFRs) or hetero-dimers (e.g. EGFR and HER-2), leading to the phosphorylation (activation) of the cytoplasmic tyrosine kinase domains. In normal cells, this leads to various cell responses including proliferation, apoptosis, migration and differentiation. Some studies suggest that during CRC, the overexpression of *EGFR* and *HER-2* genes and proteins deregulate this pathway, leading to increased cell proliferation, prolonged survival, anti-apoptosis, and metastasis [10–13]. Hence, EGFR and HER-2 are now potential targets for anticancer therapy against which cetuximab and trastuzumab, anti-EGFR and HER-2 monoclonal antibodies, have been designed and already available in market [10, 13]. In addition, there are evidences that the process of colorectal tumurogenesis may also be influenced by up regulation of cyclooxygenase-2 (COX-2; *PTGS-2* gene), the inducible form of an enzyme responsible for converting arachidonic acids into prostaglandins (PGEs) [14, 15]. PGEs play different roles in the normal physiological processes of the gastrointestinal tract, including secretion and motility, as well as pathological actions including inflammation and neoplasia. Because of these evidences, COX-2 is regarded as another potential target for the prevention of CRC; and thus, the anti-COX2 properties of potential probiotic combinations have been investigated by a number of studies [14, 16, 17].

Several studies suggest the concurrent increase in the expression of COX-2 and EGFR [18], COX-2 and HER-2 [19] and EGFR/HER-2 [20] among CRC patients. Therefore, it would be very helpful if a treatment could efficiently down regulate these onco-markers without significantly interfere with normal cells. In the present study we aimed to investigate the anti-cancer properties of some strains of *Lactobacillus* and *Bifidobacterium* spp. *in vitro* and *in vivo*.

## Materials and methods

### Treatment groups

The anti-cancer effects of the 5 following treatment groups were investigated: 1. a single strain of *Bifidobacterium*. *breve*, 2. a single strain of *Lactobacillus*. *reuteri*, 3. a cocktail of 5 strains of Lactobacilli (LC), 4. a cocktail of 5 strains of Bifidobacteria (BC), 5. a cocktail of 10 strains from both *Lactobacillus* and *Bifidobacterium* (L+B).

The five *Lactobacillus* strains were from *L*. *plantarum*, *L*. *rhamnosus*, *L*. *brevis*, and *L*. *reuteri* species and the five Bifidobacterium strains were from *B*. *bifidum*, *B*. *breve*, *B*. *longum* species (1 *B*. *longum* strain, 2 *B*. *bifidum*, and 2 *B*. *breve* strains). The *Bifidobacterium* strains were isolated in our previous study from healthy mother's milk and their healthy infants’ stool [21] and the *Lactobacillus* strains, also isolated from our previous study [22] were obtained from stool of healthy individuals. The strains were selected according to our previous results investigating the potential probiotic properties of these strains [21–23]. All the strains were kept in -80°C inside Man, Rogosa and Sharpe (MRS) broth with 20% glycerol for long-term use.

Although MRS is the medium which contains animal origin substances, this medium was only used for experimental analysis in this study and should be replaced by human compatible medium for production.

### Cell lines

The anticancer properties of the bacterial groups were assessed on 2 cell lines including the human colon adenocarcinoma cell line LS174T and the rat normal non-transformed intestinal cell line IEC-18 [24] (as the control cell line). These cells were purchased from the Cell Bank of Pasteur Institute of Iran. The cells were maintained under a humidified atmosphere with 5% CO_2_ at 37°C in high glucose DMEM medium supplemented with 10% FBS and 1% penicillin-streptomycin (Sigma‐Aldrich, UK).The reason for choosing LS174T as the colon cancer cell line was based on the previous studies reporting simultaneously high expression levels of EGFR, HER2 and COX-2 in this cell line [11, 15, 25].

### Preparation of bacterial cocktail and bacterial treatment on cells

All the bacterial strains were cultured in MRS broth (Sigma‐Aldrich, UK) under anaerobic condition at 37°C for 16 hours. The strains were then centrifuged at 8000 × g for 5 minutes and the pellets were diluted in high glucose DMEM (Thermo-Gibco,USA) containing 10% FBS (Biochrom, Berlin, Germany). The bacterial concentrations (1, 10, 100, 1000 bacteria per cell) were established by measuring the OD_600nm_ of each solution using spectrophotometer. For bacterial treatment of cells, one strain from each *Lactobacillus* and *Bifidobacterium* genera (a strain of *L*. *reuteri* and one strain of *B*. *breve*), a cocktail of 5 strains of Lactobacilli (LC), a cocktail of 5 strains of Bifidobacteria (BC), and a cocktail of the strains from both *Lactobacillus* and *Bifidobacterium* genera (L+B) were prepared. To make the bacterial cocktails equal amounts of each dilution were mixed into one tube. Cells (~3×10^5^/ well) were seeded in DMEM/ 5% FBS inside 6-well culture plates and, after 72- hours plating (allowing the cells to form tight junctions to resist the bacterial shock), the desired concentration of the bacteria (based on the results of the MTT assay) were treated onto cells and incubated for 120 hours at 37°C in 5% CO_2_.

### Cetuximab and trastuzumab anticancer drugs (study controls) and drug treatment on cells

The anti-EGFR and HER-2 monoclonal antibodies cetuximab (Erbitux; Merck, Germany) and trastuzumab (Herceptin; Aryogen Pharmad, Iran) were used as controls in this study. These drugs were diluted in sterile distilled water to prepare the concentrations of 10, 20 and 30 μg/ml for each drug. Cells (~3×10^5^/ well) were treated with the desired concentration of the drugs (based on the results of the MTT assay) and incubated for 120 hours in the same conditions as for the bacterial treatment.

### MTT anti-proliferative assay

To find the best treatment dose, the anti-proliferative effects of the bacterial and drug treatments on LS174T and IEC-18 cells were evaluated by the MTT assay using the commercially available MTT kit (Bioidea, Tehran, Iran), according to the manufacturer’s instructions. Both LS174T and IEC-18 cells (~5 × 10^3^ /well) were seeded in DMEM/ 5% FBS inside 96-well microplates and, after 24 hours plating, treated with different concentrations of bacteria (1, 10, 100, and 1000 bacteria/cell) and drugs (10, 20, and 30 μg/ml). Untreated cells were used as controls. The cells were incubated for 24 hours at 37°C in 5% CO_2_. The cell viability was then checked by reading the absorbance at 570 nm using ELISA microplate reader (BioRad, USA). The analysis for each group was repeated in triplicate. The following formula was used to calculate the percentage of proliferating cells inside each well:

$$\mathrm{Proliferating}\;\mathrm{cells}=\frac{\left(Sample\;absorbance-Blank\;absorbance\right)}{\left(Control\;absorbance-Blank\;absorbance\right)}\times 100$$

### Apoptosis assessment

Apoptosis, as a metric of evaluating anticancer properties of the bacterial groups, was determined by the Annexin V (FITC-conjugated)-Propidium iodide (PI) flow cytometric method using a commercially available kit (BioLegend, UK), according to the manufacturer’s instructions. LS174T and IEC-18 cells (~3×10^5^/ well) were seeded in DMEM/ 5% FBS inside 6-well culture plates and, after 72 hours incubation, were treated by the desired concentrations of the bacteria and drugs (based on the results of the MTT assay) and incubated for 120 hours at 37°C in 5% CO_2_. The cells were then harvested and washed twice with PBS, and following centrifugation, the cell pellets were re-suspended in Annexin binding buffer according to the manufacturer’s protocol. Cells were labeled by FITC-conjugated Annexin V and PI and analyzed on a flow cytometer (Calibur, BD Biosciences) for the detection of Annexin V and PI positive subpopulations. The experiments were performed in duplicates and the data were analyzed using the Flow Jo software. Untreated cells were used as negative controls and cetuximab and trastuzumab were used as positive controls.

### Quantitative real time PCR of the genes *EGFR*, *HER-2* and *PTGS-2*

The expression levels of *EGFR*, *HER-2* and *PTGS-2* genes, as onco-markers, were assessed using real time PCR. Briefly, the total RNA of ~10^6^ cells treated with bacterial groups, drug groups (as positive controls), as well as untreated cells (as negative controls) were extracted using a total RNA extraction kit (Roche, Germany), according to the manufacturer’s instructions. The quality and quantity of the RNAs were assessed by measuring the absorbance at 230, 260 and 280 nm in a Thermo Scientific^™^ NanoDrop 2000 and 2000c UV ⁄ Visible Spectrophotometer. RNA integrity was determined by running the samples on a 2% agarose gel. cDNA templates were synthesized from 400 ng RNA using the PrimeScript cDNA synthesis kit (Takara Bio, Japan), according to the manufacturer’s protocol. The quantification of the genes *EGFR*, *HER-2* and *PTGS-2* of the bacterial and drug- treated cells (relative to the untreated cells), were determined using SYBR Premix Ex Taq (Takara Bio, Japan) and the primers retrieved from the online Primer-Bank website http://pga.mgh.harvard.edu/primerbank (Table 1). All assays, performed in triplicate, were run in a QIAGEN Corbett rotor gene-3000. The formula RQ = 2^-ΔΔCt^ was used for the relative quantification of the transcripts. All the reactions were normalized using the glyceraldehyde 3-phosphate dehydrogenase (*gapdh*) housekeeping gene.

**Table 1:** Primer sequences used for the real time PCR of the genes *EGFR*, *HER-2* and *PTGS-2*.

Gene	Primer Bank ID	Primer Sequence (5' > 3')	Product size (bp)
***EGFR***	41327735c	F- AGGCACGAGTAACAAGCTCAC	177
		R- ATGAGGACATAACCAGCCACC	
***HER-2***	54792097c2	F- TGTGACTGCCTGTCCCTACAA	152
		R- CCAGACCATAGCACACTCGG	
***PTGS-2***	223941909c3	F-ATGCTGACTATGGCTACAAAAGC	90
		R- TCGGGCAATCATCAGGCAC	
***gapdh***	378404907c1	F-GGAGCGAGATCCCTCCAAAAT	197
		R-GCTGTTGTCATACTTCTCATGG	

### Western blotting of the proteins EGFR, HER-2 and COX-2

The expression levels of the onco-marker proteins EGFR, HER-2 and COX-2 were investigated using Western blot. Total protein was isolated from ~10^7^ cells treated with bacterial groups, drug groups (as positive controls), as well as untreated cells (as controls) using a total protein extraction kit (BioBasicInc, Canada), according to the manufacturer’s instructions. The Bradford assay was used to determine the total protein concentration, with the bovine serum albumin (BSA) (Sigma‐Aldrich, UK) used as the standard protein. The sample concentrations were equalized and diluted in Tris-Glycine-SDS sample buffer containing 2-mercaptoethanol and methylene blue (DNA biotech, Iran; cat no: DSK100) and 20 μl was loaded in a 10% sodium dodecyl sulfate polyacrylamide gel (SDS-PAGE). Lysates were resolved by electrophoresis and transferred onto PVDF membranes (Millipore, USA). The membranes were blocked for 1 hour with 5% skimmed milk in PBS/0.1% Tween-20. Membrane were immunoblotted overnight at 4°C with anti-EGFR, anti-HER-2, anti-COX-2 rabbit (Cell Signaling Technology, Inc, UK; cat no: 4267, 2165, 12282, respectively), as well as anti-rabbit monoclonal β-Actin antibody (cat no: 4970), all diluted 1:1000 in 2.5% skimmed milk in PBS/0.1% Tween-20. After washing the membrane with PBS/ 0.1% Tween-20, the membrane was incubated for 1.5 hours with horseradish peroxidase-conjugated anti-rabbit IgG (Abcam, UK; cat: ab6721), diluted 1:1000 as other antibodies. After washing, enhanced chemiluminescence (ECL; Amersham, UK) was used for detection. Each experiment was repeated in triplicate. The protein bands were quantified by the ImageJ software.

### Animals and bacterial treatments

The anticancer properties of the most effective bacterial treatment in *in vitro* experiment (BC) were further investigated *in vivo*. Female BALB/c mice (6–8 weeks old), obtained from Pasteur Institute of Iran, were kept in polycarbonate cages (5 mice per cage) under controlled environmental conditions at 22°C and 50% humidity in a 12 hours light/dark cycle. Enough food and water were made available to the animals. Mice were categorized into three groups: 1) PBS-gavaged negative control group, 2) Azoxymethane (AOM)/DSS + PBS positive control group, 3) AOM/DSS + BC in PBS test group. Five mice per group were used.

A total of 0.2 mL PBS was administered daily to the first two groups, whilst the last group was orally administered with 0.2 mL (5 × 10^11^ colony-forming units) BC in PBS. The orogastric gavage of PBS or BC was started a week before tumor induction by AOM/DSS and continued daily until sacrifice. To induce colon cancer, the second and third groups were intraperitoneally injected with a single dose of AOM (10 mg/kg) (Sigma-Aldrich) injection, as the carcinogenetic agent. After one week, water containing 2% DSS (Mpbio, United States) was given to the second and third groups for 5 days (to induce epithelial cell injury and colonic inflammation favoring the carcinogenesis process), followed by 2 weeks of recovery, replacing the DSS water with regular water. The mice received another two cycles of water containing 2% DSS [26]. The disease activity index (DAI) was determined by scoring body weight loss, stool consistency, trait of mouse activities, occult/ gross rectal bleeding, and mouse coat state and was compared between the three groups of mice.

### Ethics

All animal experiments were approved by the Animal Care and Research Advisory Committee of Iran University of Medical Sciences (ethical code: IR.IUMS.FMD.REC1396.C464) which was in accordance with the ethical standards of the Helsinki Declaration in 1975 and its later amendments.

### Tumor assessment and tissue preparation

Sixty four days after AOM injection, all mice were sacrificed by rapid cervical dislocation, excised and opened longitudinally to remove their colons. Colons were cleaned with PBS and measured for length and number of tumors from the start of ceccum to the anus. Three sections (~1 cm) of rectum samples were recovered from each mouse, fixed in 10% formalin embedded in paraffin, flash-frozen in liquid nitrogen and stored at −80°C until analyzed for histology. The colon sectionswere sectioned at 3 μm by a microtome for hematoxylin-and-eosin (H&E) staining. The slides were analyzed at a magnification of ×100. Histological score was determined using a BX43 Olympus microscope in a blind manner and possible histological variations between the three groups were compared. Because this CRC model was associated with chronic intestinal inflammation, the level of DSS-causing inflammation was also assessed and compared between the three groups of mice.

### Statistical analysis

Data were expressed as mean ± SD. For comparison between two groups Student’s t-test and Fisher exact test and for comparison between multiple groups one-way ANOVA were used in Graphpad prism Version 8. A P-value ≤ 0.05 was considered as statistically significant.

## Results

### MTT anti-proliferative assay

The MTT assay was used as an anti-proliferative assay to find the best treatment dose for the bacterial and drug groups for treating the cell lines. Untreated cells were used as controls. According to the results of the MTT assay (S1 Fig), performed in triplicates, the “no. of bacteria/ no. of cells’ ratio of 100 was chosen as the criterion for the bacterial treatment of LS 174T and IEC-18 cells since, despite a relatively high dose (ratio) of the bacteria, no more than 50% of the cells were killed in this ratio. The same ratio was used for the bacterial treatment of the IEC-18 cells, as for comparison. Concentration of 20μg/ml was chosen for cetuximab and trastuzumab for treating both cell lines since more than 50% of the cells remained intact despite a relatively high dose.

### Apoptosis assessment

Apoptosis rate was assessed by flow cytometry as a metric of evaluating anticancer properties of the bacterial groups in this study. Untreated cells were used as negative controls and cetuximab and trastuzumab were used as positive controls.

#### Effect on LS174T cells

**Fig 1:**
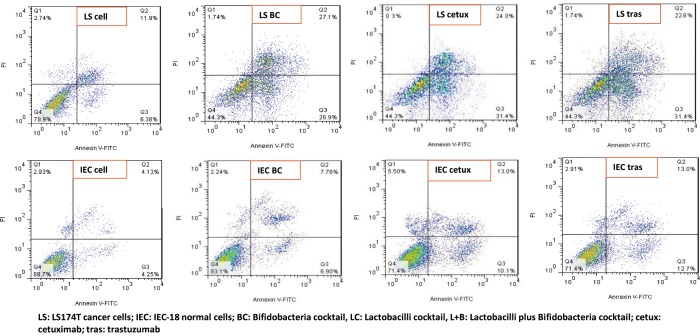
Flow cytometry analysis of cancer LS174T and normal IEC-18 cells before and after 120h treatment with bifidobacteria cocktail (100 bacteria/cell), cetuximab and trastuzumab. Cells considered as viable were Annexin V and PI negative; cells in early apoptosis stage were Annexin V positive and PI negative; and cells in late apoptosis/ necrosis stage were both Annexin V and PI positive. Untreated cells were used as negative controls and cetuximab and trastuzumab were used as positive controls.

The flow cytometry results, performed in duplicates, showed that among the bacterial treatments, BC (20.5% primary apoptosis) had the highest effect on apoptosis induction after 120h incubation on LS174T cells (P = 0.03) (Fig 1).

LC, *B*. *breve*, L+B and *L*. *reuteri* induced 18.52%, 17.12%, 16.62%, and 11.12% primary apoptosis on LS174T cells after 120h, respectively (S2 Fig).

Cetuximab and trastuzumab respectively induced 25.02% and 15.3% apoptosis among LS174T cells.

The flow cytometry data showed that both Bifidobacteria and Lactobacilli act better to induce apoptosis when in cocktail preparations, rather than single strain treatments.

The apoptosis rate was lower among IEC-18 cells, compared to LS174T cells for all treatment groups (S2 Fig).

BC (93.7% survival and ~3% apoptosis rate) managed to have the least interruption on IEC-18 cells (P = 0.05).

L+B, *B*. *breve*, *L*. *reuteri* and LC respectively induced 8.75%, 8.45%, 4.61% and 4.31% higher apoptosis rates among IEC-18 cells, compared to untreated control cells.

Cetuximab and trastuzumab respectively induced 5.85% and 8.45% apoptosis among IEC-18 cells.

### Quantitative real time PCR of the genes *EGFR*, *HER-2* and *PTGS-2*

Real time PCR was used to assess the expression levels of *EGFR*, *HER-2* and *PTGS-2* genes, as onco-markers in colorectal cancer. In all assessments, untreated cells were used as negative controls and cetuximab and trastuzumab were used as positive controls. All the experiments were carried out in triplicates.

#### *EGFR* expression among LS174T cells

**Fig 2:**
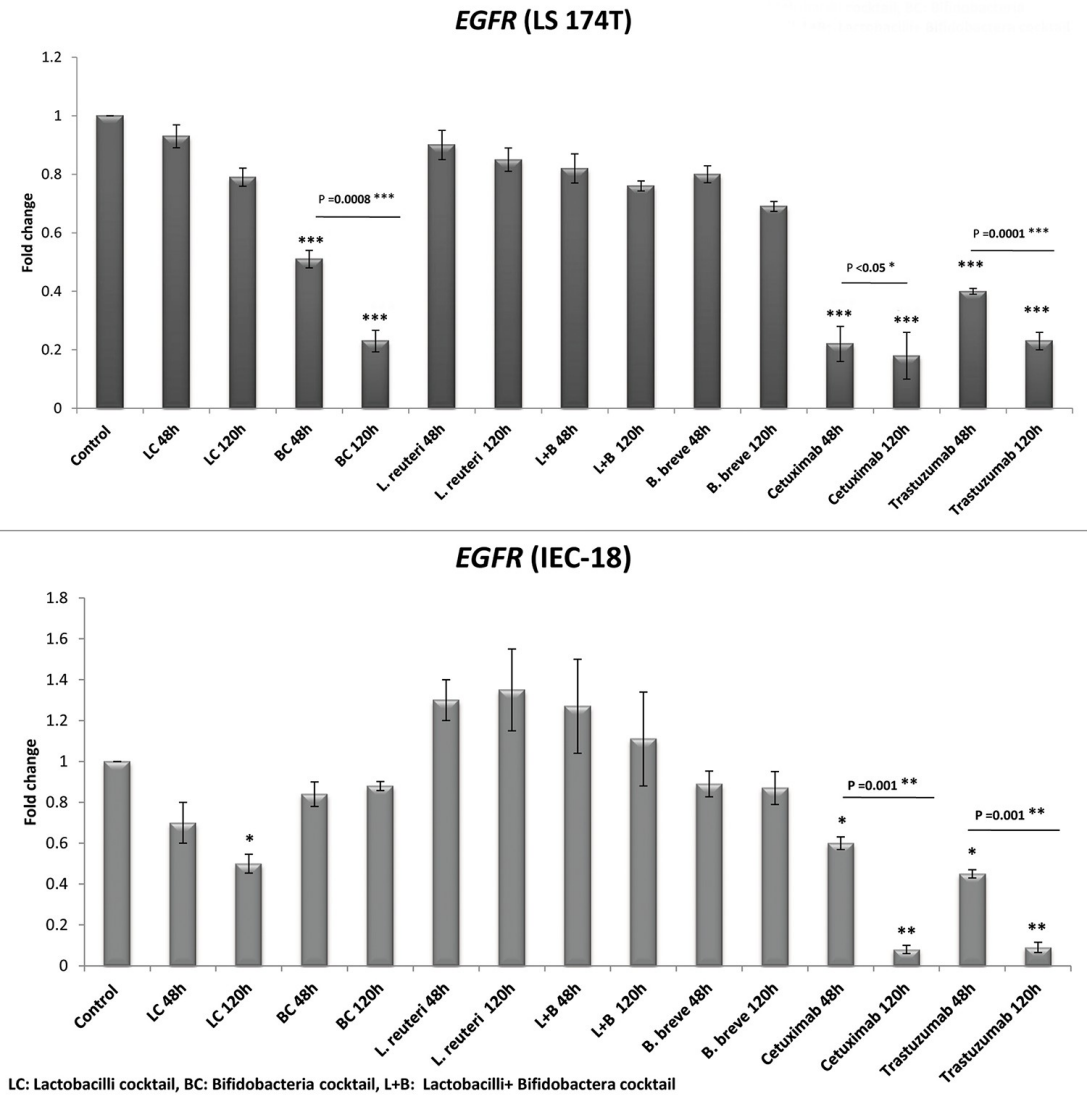
Relative fold change (relative to untreated control cells) of the EGFR gene among LS174T and IEC-18 cells. Results were expressed as mean; error bars (SD); n = 3. Statistical analysis was performed using one-way ANOVA test. * indicates P-values less than 0.05, ** indicates P-values less than 0.01, and *** indicates P-values less than 0.001. Untreated cells were used as negative controls and cetuximab and trastuzumab were used as positive controls.

The results presented in Fig 2 demonstrate that, compared to the control cells, all the bacterial groups down regulated the *EGFR* gene among LS174T cells during 120h incubation. However, the results were only significant in case of BC (4.4 folds decrease, P = 0.0001), as was also the case for cetuximab and trastuzumab (5.6 and 4.4 folds decrease, respectively; P = 0.0001).

There was no significant difference between the effectiveness of the other 4 bacterial treatments in *EGFR* down regulation (*B*. *breve*, L+B, LC, and *L*. *reuteri* respectively decreased *EGFR* expression by 1.5, 1.3, 1.3, 1.2 folds).

#### *EGFR* expression among IEC-18 cells

Most bacterial groups decreased the *EGFR* expression among the IEC-18 normal cells (1.2, 1.2, and 2 folds decrease for BC, *B*. *breve* and LC, respectively), whilst 2 groups increased its expression (1.35 and 1.11 folds increase for *L*. *reuteri* and L+B, respectively). These changes were only significant in case of LC (2 folds decrease, P<0.05), as was also the case for cetuximab and trastuzumab (12.5 and 11.1 folds decrease, respectively, P = 0.009).

#### *HER-2* expression among LS174T cells

**Fig 3:**
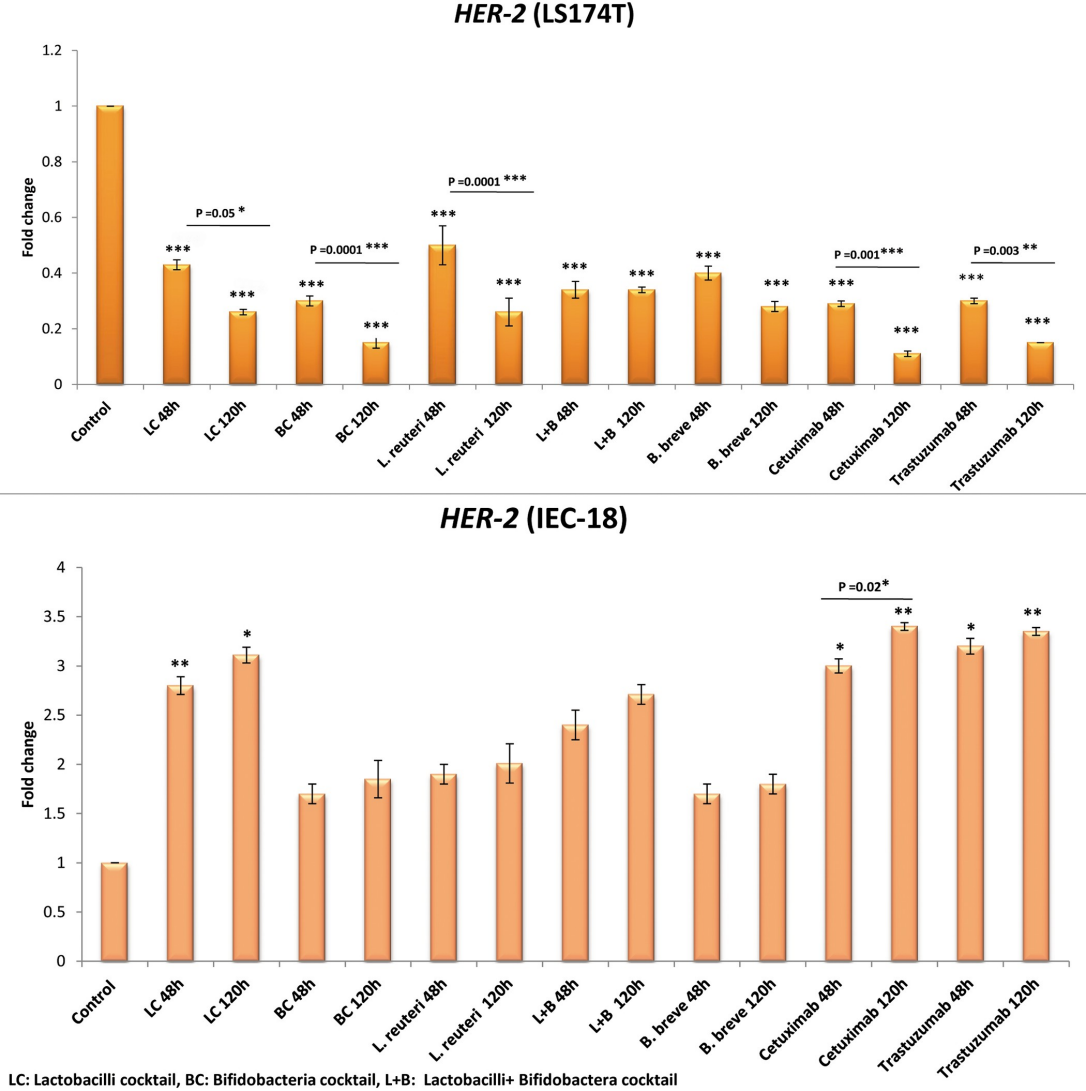
Relative fold change (relative to untreated control cells) of the *HER-2* gene among LS174T and IEC-18 cells. Results were expressed as mean; error bars (SD); n = 3. Statistical analysis was performed using one-way ANOVA test. * indicates P-values less than 0.05, ** indicates P-values less than 0.01, and *** indicates P-values less than 0.001. Untreated cells were used as negative controls and cetuximab and trastuzumab were used as positive controls.

Comparing Figs 2 and 3 shows that the effects of the studied bacterial groups in down regulating *HER-2* was much more pronounced among LS174T cells, compared to the *EGFR* gene. The reduction in the expression of *HER-2* was significant for all the bacterial groups (P = 0.0001), similar to the drug groups (P = 0.001).

BC down regulated *HER-2* by 6.7 folds but there was no significant difference between the effectiveness of all the other 4 bacterial groups in *HER-2* down regulation (LC, *L*. *reuteri*, *B*. *breve*, and L+B decreased *HER-2* expression by 3.9, 3.9, 3.6, 3 folds, respectively).

Cetuximab and trastuzumab respectively down regulated *HER-2* by 9.1 folds (P = 0.05) and 6.7 folds (P = 0.09).

#### *HER-2* expression among IEC-18 cells

All the bacterial groups increased *HER-2* expression among the IEC-18 normal cells. This increase was only significant in case of LC (3.11 folds increase; P<0.01), as was also the case for cetuximab and trastuzumab (3.4 and 3.35 folds increase, respectively; P = 0.001).

#### *PTGS-2* expression among LS174T cells

**Fig 4:**
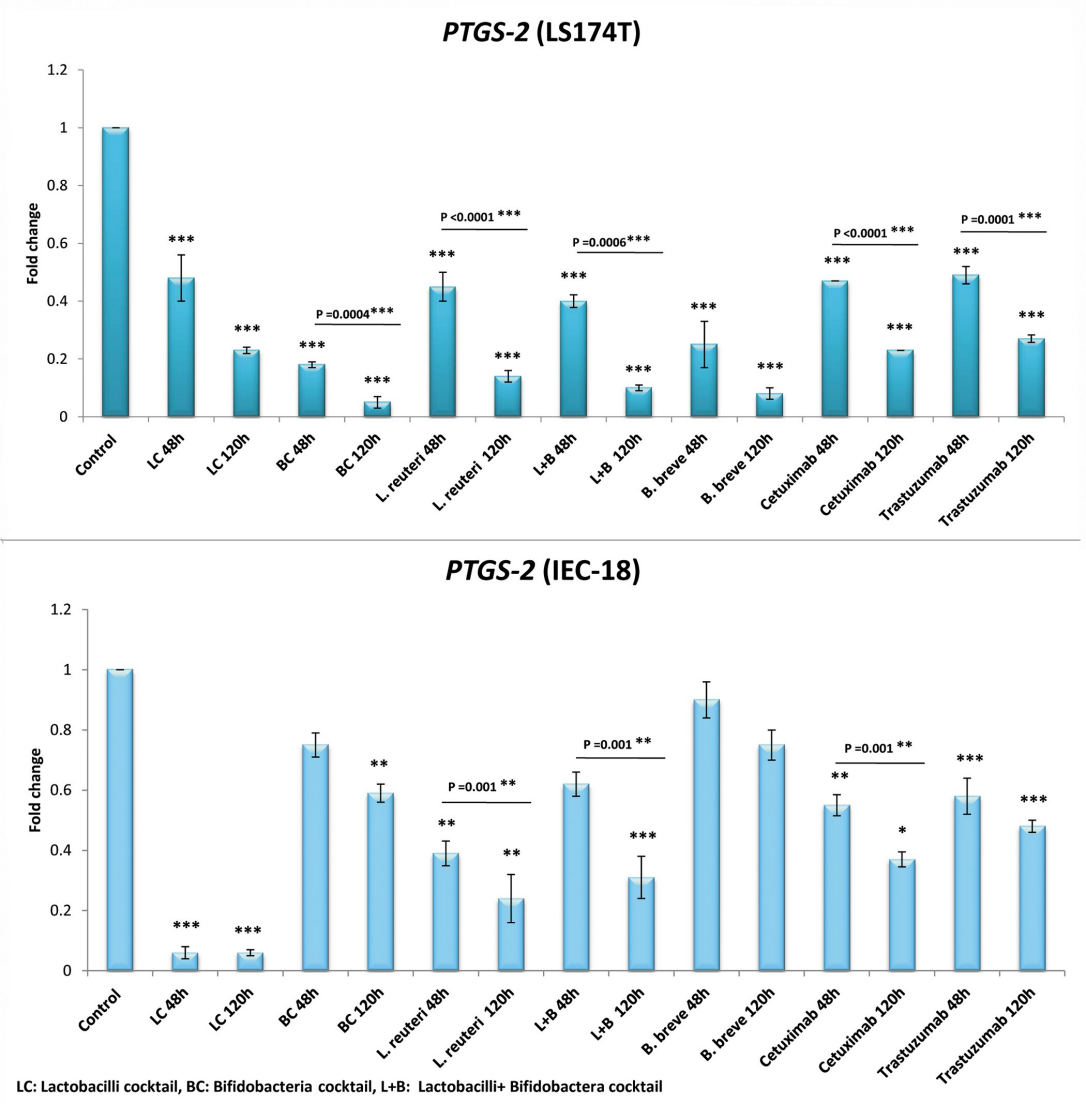
Relative fold change (relative to untreated control cells) of the *PTGS-2* gene among LS174T and IEC-18 cells. Results were expressed as mean; error bars (SD); n = 3. Statistical analysis was performed using one-way ANOVA test. * indicates P-values less than 0.05, ** indicates P-values less than 0.01, and *** indicates P-values less than 0.001. Untreated cells were used as negative controls and cetuximab and trastuzumab were used as positive controls.

Fig 4 shows that the expression of *PTGS-2* among LS174T is more considerably influenced by the bacterial groups, compared to the *EGFR* and, to a less extent, to the *HER-2* genes. A very significant reduction is observed in the expression of *PTGS-2* compared to the control, for all the bacterial groups (P<0.0001), similar to the cetuximab and trastuzumab (4.4 and 4 folds decrease in *PTGS-2* expression, P = 0.005).

BC (20 folds decrease in *PTGS-2* expression) had the highest influence on down regulating *PTGS-2*, among the bacterial groups.

*B*. *breve*, L+ B, *L*. *reuteri* and LC reduced *PTGS-2* expression by 12.5, 10, 9.1 and 4.4 folds, respectively.

#### *PTGS-2* expression among IEC-18 cells

All the bacterial groups, except *B*. *breve*, significantly decreased the *PTGS-2* expression among the IEC-18 normal cells (1.7, 3.2, 4.2, 16.7 folds decrease for BC, L+B, *L*. *reuteri* and LC, respectively; P = 0.05). *B*. *breve*, although decreased *PTGS-2* expression (by 1.3 folds), its result was not significant.

Cetuximab and trastuzimab both decreased *PTGS-2* expression by 4.4 folds.

### Western blotting of the proteins EGFR, HER-2 and COX-2

**Fig 5:**
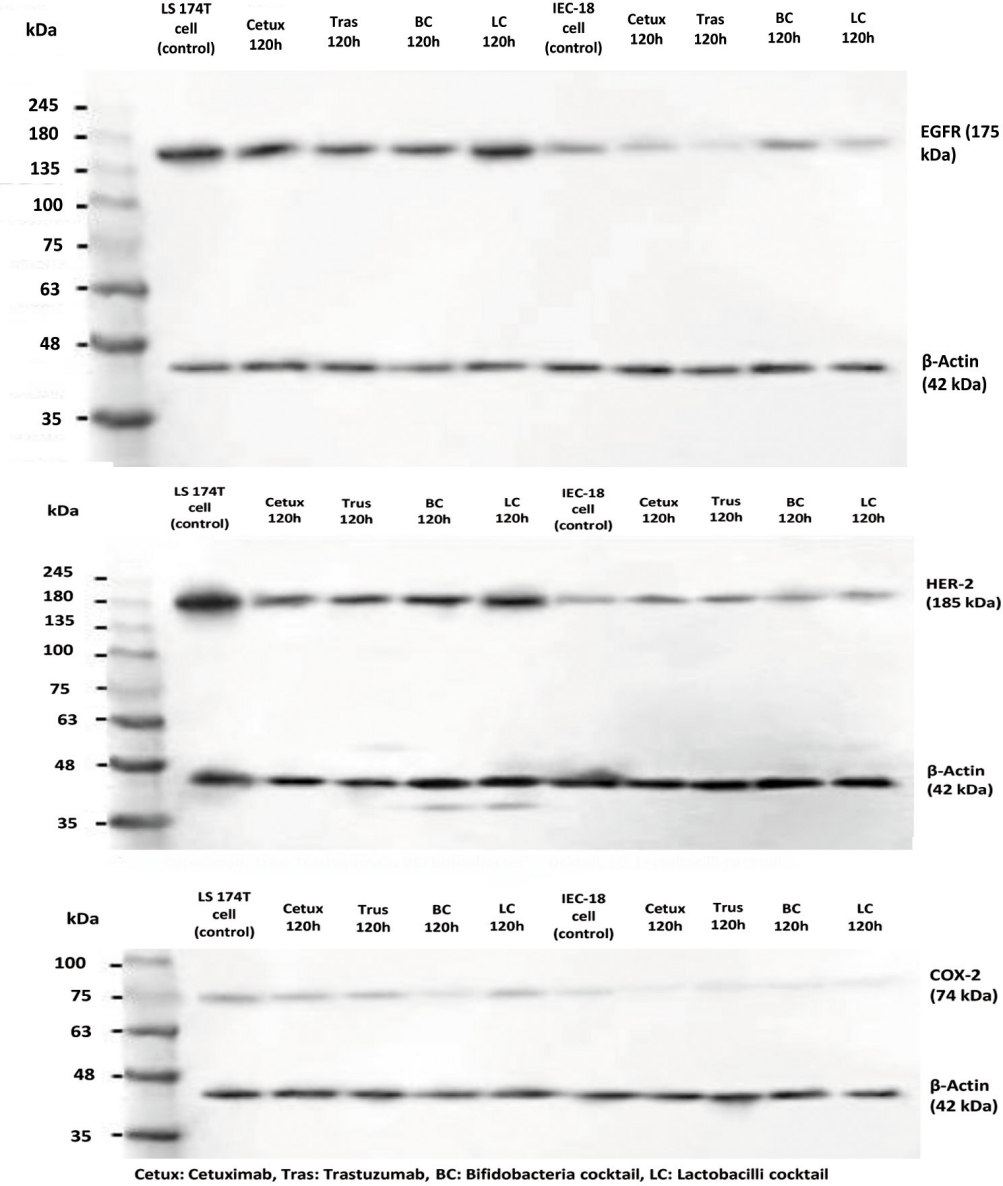
Western blot analysis demonstrating the expression of EGFR, HER-2 and COX-2 proteins in LS174T and IEC-18 cells. Untreated cells were used as negative controls and cetuximab and trastuzumab were used as positive controls. β-Actin was used as the loading control.

Western blot analysis was performed to evaluate the expression levels of the proteins EGFR, HER-2 and COX-2. Untreated cells were used as negative controls and cetuximab and trastuzumab were used as positive controls. All the experiments were performed in triplicates. Western blotting (Fig 5, S1 Table) showed that BC significantly reduced EGFR, HER-2 and COX-2 protein levels among LS174T cells, as did cetuximab and trastuzumab (P = 0.002). On the other hand LC, although reduced the expression of these proteins, was significantly less efficient than BC (P = 0.01).

BC had no significant changes in the expression of these proteins among IEC-18 cells and the effect of LC among IEC-18 cells was only significant in case of COX-2 levels.

Both drugs significantly changed the expression of EGFR, HER-2 and COX-2 among IEC-18 cells (P = 0.001).

### Animal treatments

**Fig 6:**
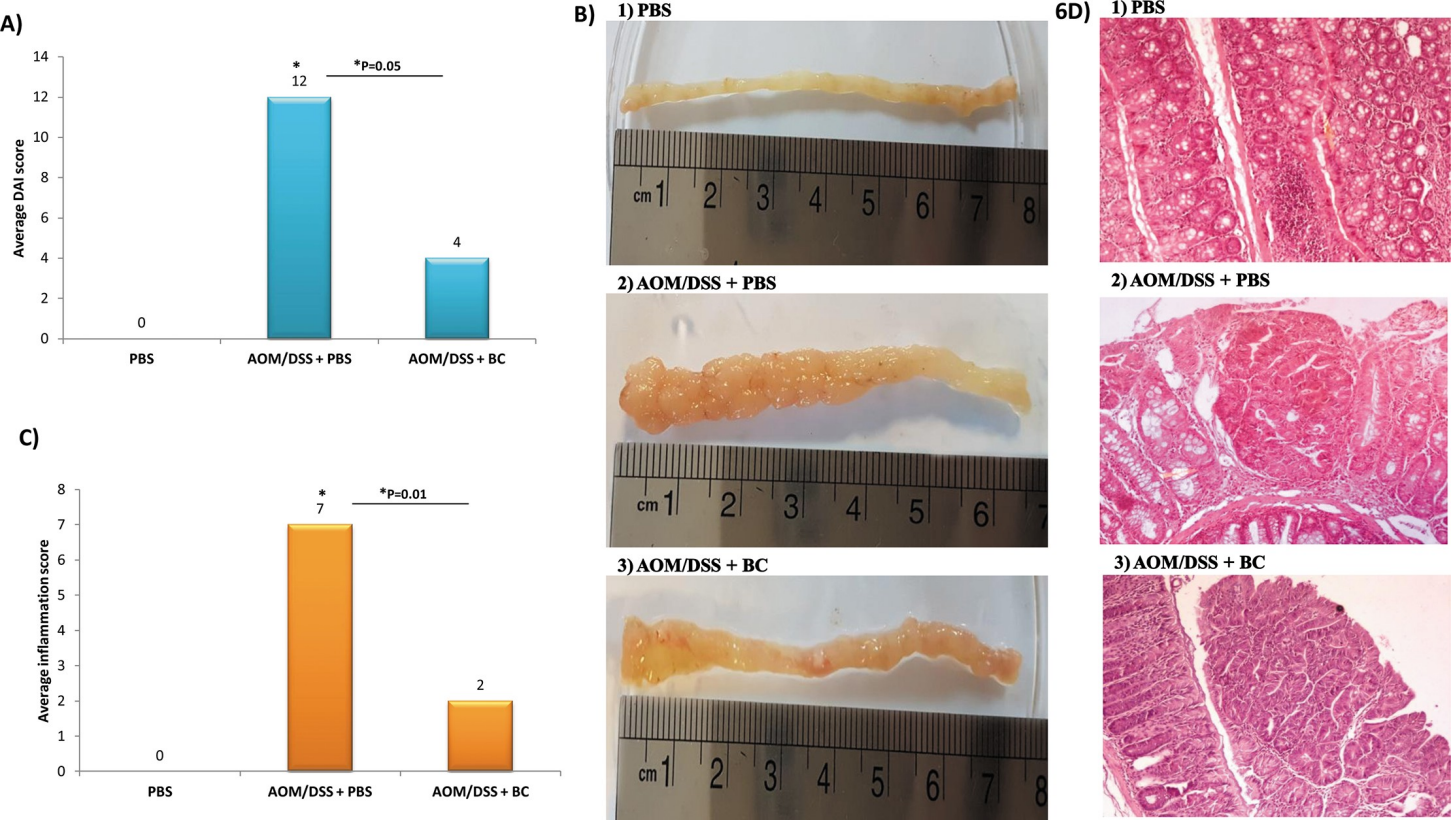
Macroscopic and histological assessment of colic tumor and inflammation in mice. A) Average Disease Activity Index (DAI) score, B) Colonic length and macroscopic tumor incidence, C) Semi-quantitative scoring of average histological inflammation, D) Representative H&E-stained images of distal colon tissues from 1) PBS mice group (negative control), AOM/DSS + BC group and 3) AOM/DSS + PBS mice (positive control); scale bars, 200 μm. Data were represented as the mean ± SD (*n* = 5 mice per group).

BC was the most effective treatment *in vitro*, and therefore its anticancer properties were further investigated in mice models. As shown in Fig 6A, The AOM/DSS + BC (third group) had a significantly lower DAI, compared to the AOM/DSS-induced CRC mice (second group) (P = 0.05).

Tumors presented in BC-treated mice were also significantly smaller than those observed in untreated CRC mice (P = 0.001). BC-treated mice had significantly higher colon length and lower tumors incidence (average length: 7.5cm, average number: 4), compared to AOM/DSS CRC mice (average length: 6.4, average number: 31) (P = 0.04) (Fig 6B). Histological assessments showed that colon sections of the mice fed with BC showed significantly lower inflammation (Fig 6C) (P = 0.01), tumor stage and grade (G1 low grade) compared to the AOM/DSS CRC mice group (G2 low grade) (Fig 6D) (P = 0.04).

## Discussion

Probiotics have recently been suggested as interesting cancer preventive/ treating bacteria which exert their anti-cancer properties through different proposed mechanisms. Some limited studies have suggested interference of certain probiotics with the activation of EGFR family and COX-2 expression, the over expression of which are involved in many cancer types [16, 17, 27–29]. Several studies suggest the concurrent increase in the expression of COX-2 and EGFR [18], COX-2 and HER-2 [19] and EGFR/HER2 [20] among CRC patients. Therefore, it would be very useful if a treatment could efficiently down regulate these onco-markers without significantly interfere with normal cells. We had previously demonstrated that five *Lactobacillus* spp., from normal fecal samples, and five *Bifidobacterium* spp., from mother’s milk and infant stool, had different degrees of anti-proliferative, anti-pathogenic, antibacterial and antimicrobial activities [21, 23].

In this study, BC (20.5% primary apoptosis) had the highest effect on inducing apoptosis after 120h incubation on LS174T cells among the bacterial treatments. Besides, BC managed to have the least interruption on IEC-18 cells (~3% apoptosis rate). All these results prove the very effective apoptotic feature of BC compared to other bacterial treatments, with a “protective” property despite a high ability of apoptosis induction in cancer cells.

LC and *B*. *breve* were the next functional candidates among the potential probiotics tested, which had reasonable apoptotic effects on LS174T cells whilst being relatively benign on IEC-18 cells.

Several other studies have also confirmed the apoptotic effects of different strains of probiotics in cancer cells and animal models [4, 7, 30, 31]. It has been shown that *Lactobacillus* and *Bifidobacterium* spp. are able to change the expression of genes involved in cell death, apoptosis, metastasis, and cell proliferation by several studies [6]. Tiptiri-Kourpeti et al demonstrated that *Lactobacillus casei* ATCC 393 induced apoptosis of colon carcinoma cells by up regulating the tumor necrosis factor-related apoptosis-inducing ligand (TRAIL) protein which, in turn, decreased tumor incidence in mice [5]. In addition, Patricia W Lin et al. have confirmed an anti-apoptotic mechanism for the probiotic *L*. *rhamnosus GG* on normal IEC-18 cell line [32].

Moreover, BC had a notable result among other bacterial groups with similar effect as cetuximab (25.02% primary apoptosis) but had a significantly higher influence than trastuzumab on apoptosis induction on LS174T cells after 120h incubation (P = 0.001). In addition, all of the bacterial groups, except *L*. *reuteri*, were more effective than trastuzumab (15% primary apoptosis) in terms of apoptosis induction on LS174T cells and all, except BC, had generally less apoptotic activity than cetuximab.

Since cetuximab and trastuzumab bind to the outer membrane section of EGFR and HER-2 proteins, it is believed that the signaling responses triggered by activation of these two receptors result in apoptosis of cancer cells [33–35].

It might be that the signaling pathways triggered by cetuximab- EGFR binding and BC- cell attachment are equally strong in apoptosis induction. The reason of higher apoptotic effect of bacterial groups compared with trastuzumab could be that they affect multiple signaling pathways concomitantly (in addition to HER-2 and EGFR pathways) for apoptosis induction which make the total triggered signal stronger than trastuzumab. In addition, bacteria as living organisms have the ability of quorum sensing their environment, which probably help them synergistically activate stronger apoptotic signals in the cell. It may prove that a combination of Bifidobacterial strains have a stronger quorum sensing, compared to other tested bacteria, in response to attachment to cancer cells and hence, the higher apoptotic activity of BC compared to other bacterial groups.

The low expression of EGFR and HER-2 receptors in normal cells compared to LS174T cells [11, 36] (as also confirmed by the western blotting and real time analysis in our study), could be the reason for the low apoptosis rate by the treatment groups in IEC-18 cells through a lower amount of drug/bacteria-receptor attachment in these cells.

In general, the apoptosis rate induced by the bacterial groups does not seem to be merely dependent on the cells intrinsic characteristics (e.g. the number and types of receptors on the cells) but also on the characteristics of the bacterial treatments since, for instance, BC had the highest apoptosis rate in LS174T cells compared to other bacteria, but the lowest rate in IEC-18 cells. One of these bacterial characteristics could be surface proteins and the attaching power to different receptors which are different among bacteria.

In this study, BC had significant EGFR-down regulating effects among LS174T cells, both at mRNA and protein levels, whilst having the least interruption on IEC-18 cells compared to other treatments (in terms of down regulating this onco-marker). These properties make BC a very efficient anti-EGFR treatment which significantly reduces EGFR in cancer cells whilst having no significant impact (regarding EGFR levels) on normal cells. LC, on the other hand, had no significant effect on LS174T in terms of reducing the EGFR expression (neither on the mRNA nor the protein levels), but significantly decreased the gene expression in normal cells during 120h incubation time; therefore, LC seems not efficient in this regard.

The rationale for all this should again come from the fact that the plethora of surface proteins differ among bacteria/ and strains of bacteria which makes their attaching power to variant cell types different.

There were no preferences between the three bacterial treatments in decreasing *EGFR* levels. One result to be noted is that all the bacterial groups had significantly less interruptions in the *EGFR* expression among normal IEC-18 cells, compared to cetuximab and trastuzumab. Since Lactobacilli and Bifidobacteria are the commensal flora of the gut in many living organisms, it seems logical that they have less interruptions in normal epithelial cells compared to the drugs.

Compared to EGFR, HER-2 (mRNA and protein levels) was much more efficiently inhibited by the bacterial groups in LS174T cells. Several receptors and factors on the cell might be affected by these bacteria that all end up influencing the expression of HER-2 in the cell. Among the ErbB family receptors, HER-2 (ErbB-2) has a higher capacity to form heterodimers with other family members (ErbB1/ErbB2, ErbB2/ErbB3, and ErbB2/ErbB4) (https://www.kegg.jp). If we assume that the signals triggered by the heterodimers finally regulate the expression of both members of the heterodimer compartment, then the bacteria might have a higher chance of down regulating HER-2 (compared to EGFR/ErbB-1) in the cell. BC was the strongest bacterial treatment in down regulating HER-2 in LS174T cells (the same strength as trastuzumab and only second to cetuximab) but did not significantly change this tumor marker’s expression among IEC-18 cells (in contrast to the drugs). LC had a good effect on cancer cells in terms of down regulating HER-2, but it significantly increased the expression of HER-2 among IEC-18 cells (as did the drugs). Therefore, LC might again have anti-HER2 properties of questionable value. The reason why HER-2 expression among IEC-18 cells has unexpectedly increased by the treatment groups in this study remains unclear.

The bacterial groups had a higher effect on down regulation of COX-2 (mRNA and protein levels) compared to EGFR and, to a lesser extent, to HER-2. BC, with 20 folds decrease in *PTGS-2* expression, was again the most effective bacterial treatment in *PTGS-2* down regulation among LS174T cells (acted ~ 5 folds more effective than the drugs). In the meanwhile, BC did not have a notable effect on *PTGS -2* expression among normal IEC-18 cells (significantly less interruptive than the drugs). For all these reasons, BC was again the most effective treatment, compared to other bacterial groups, in decreasing COX-2 in cancer cells while not having a great impact on this gene among normal cells.

Another interesting remark in this study was that all the bacterial groups, except LC, were stronger in *PTGS-2* down regulation among LS174T cells, compared to cetuximab and trastuzumab. This might be an expected observation since the *PTGS-2* down regulating effects of some probiotic strains have already been reported by several studies [16, 17], whilst the main mechanisms of action for cetuximab and trastuzumab are the blockage of EGFR and HER-2 pathways and not the COX-2 expression. Since these two drugs are used as the comparative measures (against potential probiotics) for assessing changes in EGFR and HER-2 expressions, it would have been interesting to see whether or not they have any impact on the expression of *PTGS-2* gene and COX-2 protein. Besides, there are studies which have reported an association between the expression of COX-2 with HER-2 [15] and EGFR [37], as well with cetuximab resistance [38]. Cetuximab and trastuzumab were both able to significantly reduce the expression of *PTGS-2* gene in LS174T and also in IEC-18 cells. This might suggest an indirect influence of cetuximab and trastuzumab on COX-2 expression and might affect the signaling pathways in which COX-2 is involved.

The beneficial effects of BC on CRC- prevention was proved by *in vivo* experiments in this study. BC significantly ameliorated the DAI, almost completely restored the colon length, inhibited the increase in tumor incidence and prevented the progress of tumors to higher stages and grades.

Studies by Le Leu et al. showed that the probiotic *Bifidobacterium lactis* synbiotically combined with resistant starch significantly prevented CRC among rat-AOM model as well as facilitating apoptotic deletion of carcinogen-damaged cells in rat colon [4, 7]. In another study, Challa et al. demonstrated that the probiotic *Bifidobacterium longum* combined with lactulose significantly suppressed AOM-induces colonic crypt foci which are preneoplastic markers [3].

All these results confirm the preventive role of BC in CRC occurrence among CRC animal models.

## Conclusions

Overall, considering the effects on both the cancer and normal cell lines, Bifidobacteria cocktail is the most efficient treatment, compared to other bacterial combinations used in this study. This potential probiotic has considerable “protective” anti-cancer properties comparable to the already in use drugs cetuximab and trastuzumab and is able to concomitantly down regulate *EGFR*, *HER-2* and *PTGS-2* (COX-2) onco-markers and significantly ameliorate disease activity index, restore colon length, inhibit the increase in tumor incidence and prevent the progress of tumors to higher stages and grades.

In general, this potential probiotic could be considered as a suitable nutritional supplement to be used along with the drugs cetuximab and trastuzumab to treat and prevent CRC.

Since the results of this study is probably strain- and cell type- specific, it is recommended that more variants of Bifidobacterial strains and cell types be investigated to obtain a more comprehensive conclusion on the anti-CRC mechanisms of action of this bacterium.

## Supporting information

S1 Fig Effects of the bacterial groups on LS 174T cancer cells (A); cetuximab and trustuzumab drugs on LS 174T cancer cells (B); bacterial groups on IEC-18 primary cells (C); cetuximab and trustuzumab drugs on IEC-18 primary cells. Results were expressed as mean; error bars (SD); n = 3. Statistical analysis was performed using one-way ANOVA test. * indicates P-values less than 0.05, ** indicates P-values less than 0.01, and *** indicates P-values less than 0.001. Untreated cells were used as controls.
(TIF)

S2 Fig Flow cytometry analysis of cancer LS174T and normal IEC-18 cells before and after treatment with potential probiotics and drugs. Cells considered as viable were Annexin V and PI negative; cells in early apoptosis stage were Annexin V positive and PI negative; and cells in late apoptosis/ necrosis stage were both Annexin V and PI positive. Untreated cells were used as negative controls and cetuximab and trastuzumab were used as positive controls.
(PDF)

S1 Table The relative expression of EGFR, HER-2 and COX-2 proteins among LS174T and IEC-18 cells. (PDF)

S1 Raw images (TIF)
