# Correction: Anti-cancer effects of *Bifidobacterium* species in colon cancer cells and a mouse model of carcinogenesis

**DOI:** 10.1371/journal.pone.0234777

**Published:** 2020-06-10

**Authors:** Parisa Asadollahi, Roya Ghanavati, Mahdi Rohani, Shabnam Razavi, Maryam Esghaei, Malihe Talebi

In the Materials and methods subsection of the Abstract, there is an error in the first sentence. The correct sentence is: Anticancer effects of the following potential probiotic groups were investigated in LS174T cancer cells compared to IEC-18 normal cells.

There are errors in the Copyright statement. The correct Copyright statement is as follows: © 2020 Asadollahi et al. This is an open access article distributed under the terms of the Creative Commons Attribution License, which permits unrestricted use, distribution, and reproduction in any medium, provided the original author and source are credited.

All six authors’ names are incorrect. The correct names are: Parisa Asadollahi, Roya Ghanavati, Mahdi Rohani, Shabnam Razavi, Maryam Esghaei, Malihe Talebi. The correct citation is: Asadollahi P, Ghanavati R, Rohani M, Razavi S, Esghaei M, Talebi M (2020) Anti-cancer effects of Bifidobacterium species in colon cancer cells and a mouse model of carcinogenesis. PLoS ONE 15(5): e0232930. https://doi.org/10.1371/journal.pone.0232930
