# Correction: Anti-cancer effects of *Bifidobacterium* species in colon cancer cells and a mouse model of carcinogenesis

**DOI:** 10.1371/journal.pone.0242387

**Published:** 2020-11-19

**Authors:** Parisa Asadollahi, Roya Ghanavati, Mahdi Rohani, Shabnam Razavi, Maryam Esghaei, Malihe Talebi

Figs 2, 3, and 4 are incorrect. The figures contain information that does not correspond to the results presented in the article. The authors have provided corrected versions here.

**Fig 2:**
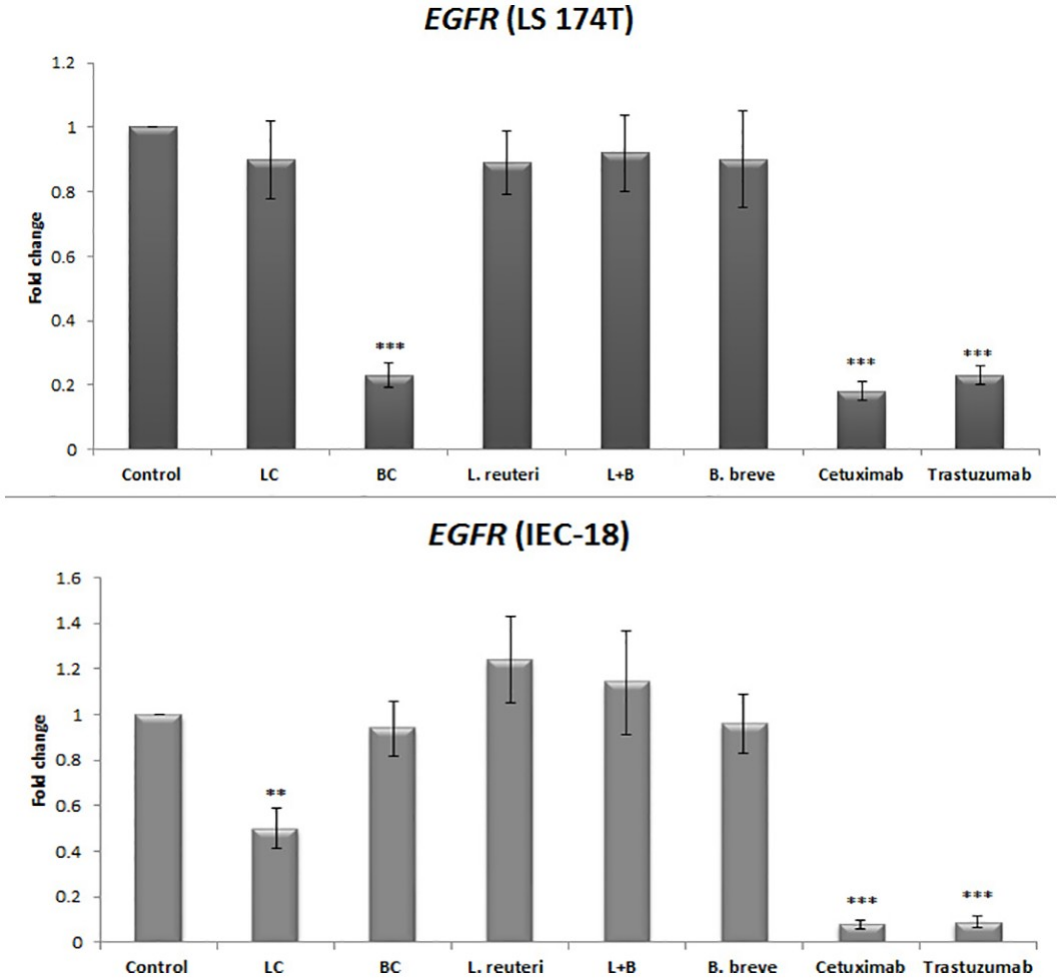
Relative fold change (relative to untreated control cells) of the *EGFR* gene among LS174T and IEC-18 cells. Results were expressed as mean; error bars (SD); n = 3. Statistical analysis was performed using one-way ANOVA test. * indicates P-values less than 0.05, ** indicates P-values less than 0.01, and *** indicates P-values less than 0.001. Untreated cells were used as negative controls and cetuximab and trastuzumab were used as positive controls.

**Fig 3:**
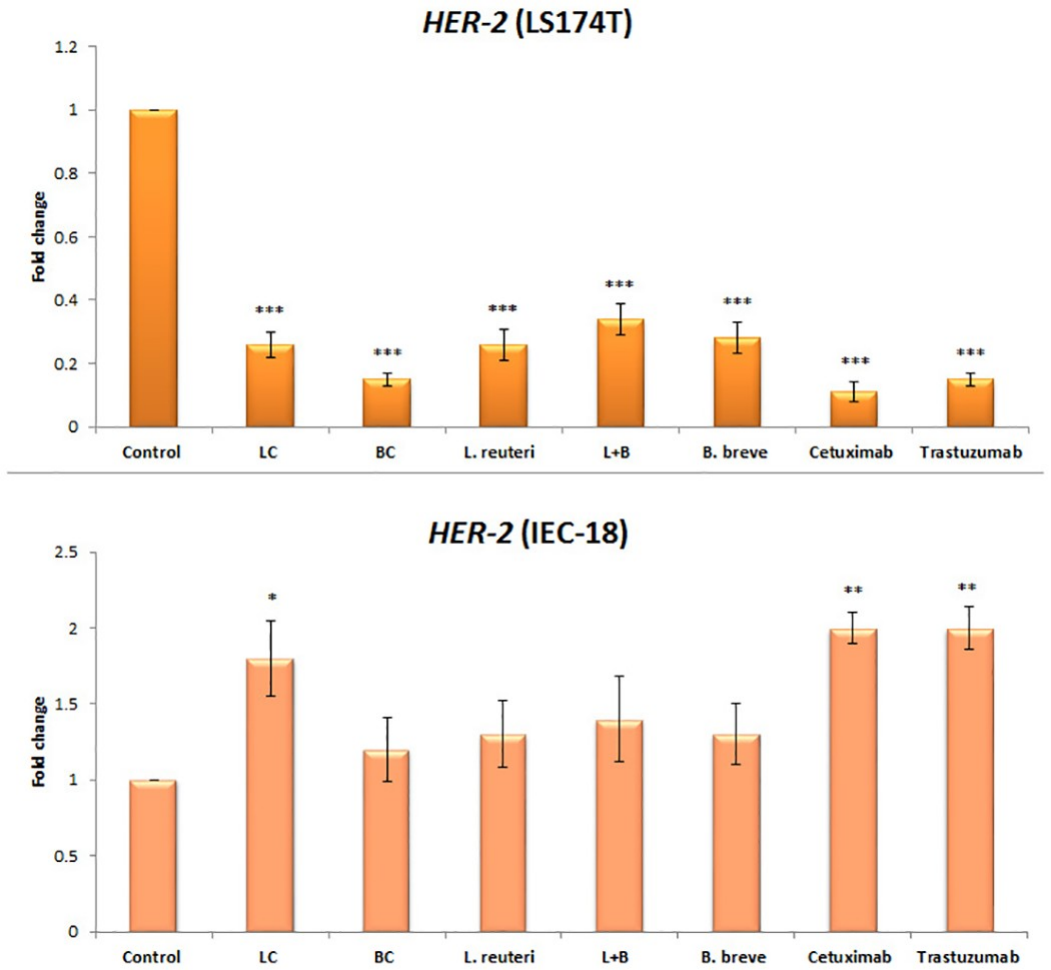
Relative fold change (relative to untreated control cells) of the *HER-2* gene among LS174T and IEC-18 cells. Results were expressed as mean; error bars (SD); n = 3. Statistical analysis was performed using one-way ANOVA test. * indicates P-values less than 0.05, ** indicates P-values less than 0.01, and *** indicates P-values less than 0.001. Untreated cells were used as negative controls and cetuximab and trastuzumab were used as positive controls.

**Fig 4:**
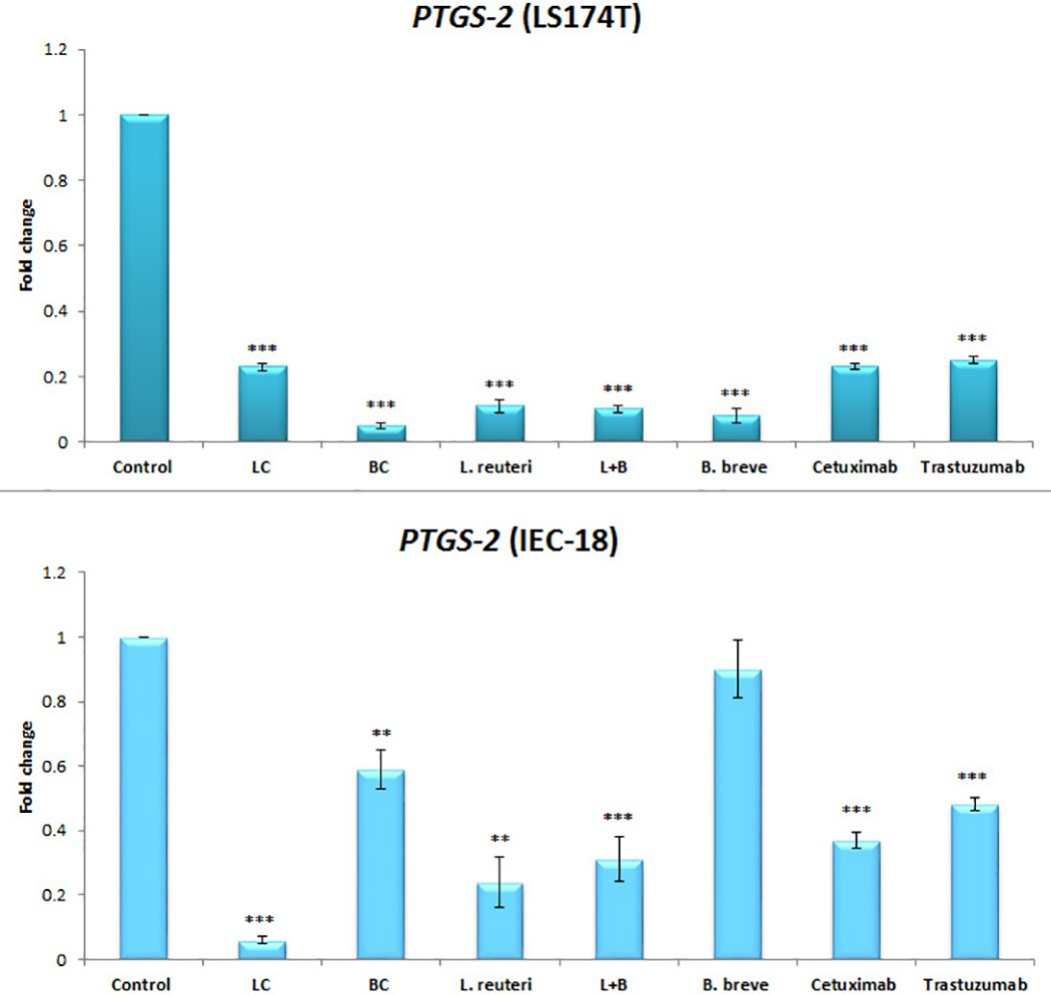
Relative fold change (relative to untreated control cells) of the *PTGS-2* gene among LS174T and IEC-18 cells. Results were expressed as mean; error bars (SD); n = 3. Statistical analysis was performed using one-way ANOVA test. * indicates P-values less than 0.05, ** indicates P-values less than 0.01, and *** indicates P-values less than 0.001. Untreated cells were used as negative controls and cetuximab and trastuzumab were used as positive controls.
