# Supplementary figures and images for: Anti-cancer effects of Bifidobacterium species in colon cancer cells and a mouse model of carcinogenesis
Source: PLoS One. 2020 May 13;15(5):e0232930. doi: 10.1371/journal.pone.0232930 (PMC7219778; doi:10.1371/journal.pone.0232930)

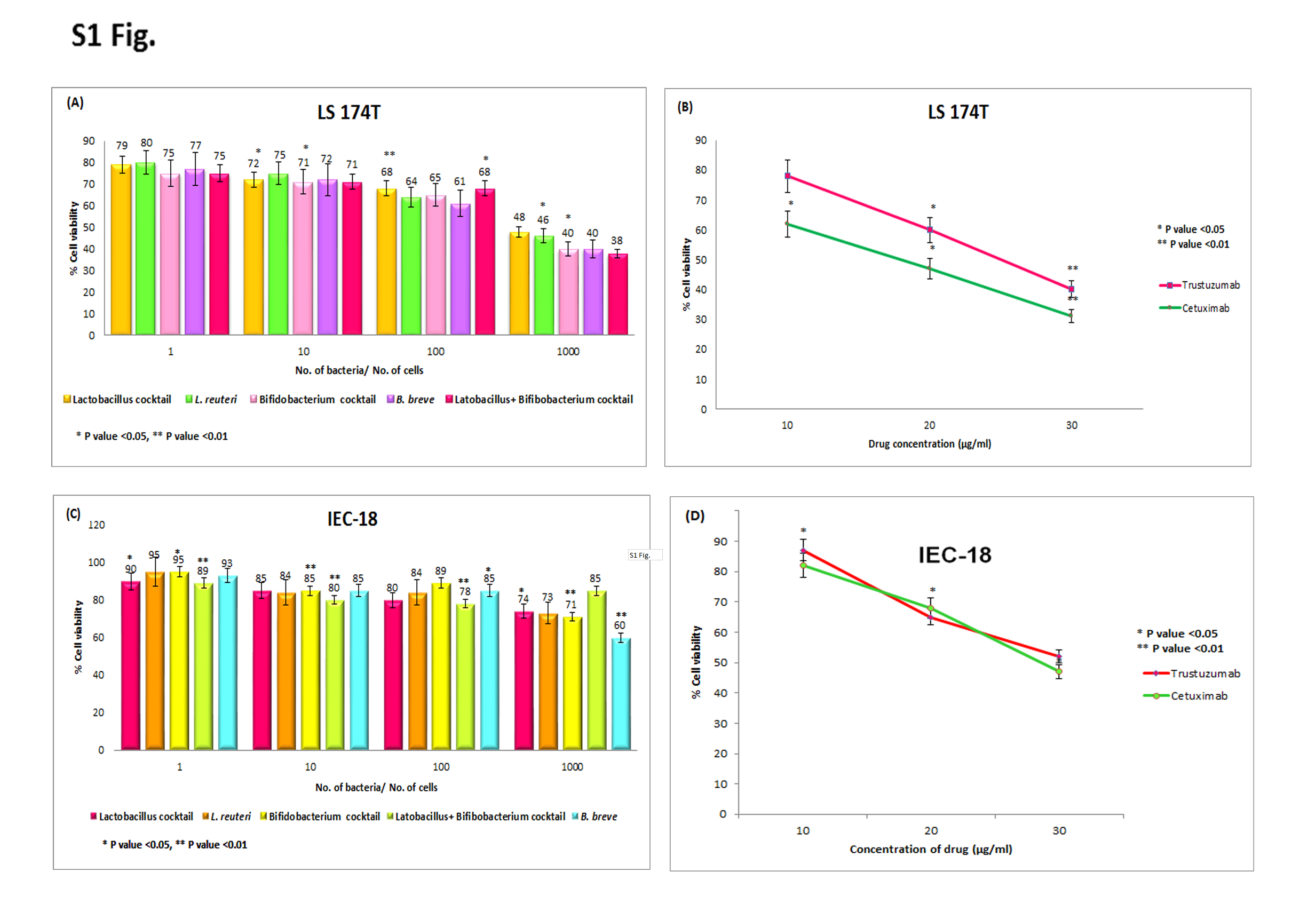

Supplement: S1 Fig — Effects of the bacterial groups on LS 174T cancer cells (A); cetuximab and trustuzumab drugs on LS 174T cancer cells (B); bacterial groups on IEC-18 primary cells (C); cetuximab and trustuzumab drugs on IEC-18 primary cells. Results were expressed as mean; error bars (SD); n = 3. Statistical analysis was performed using one-way ANOVA test. * indicates P-values less than 0.05, ** indicates P-values less than 0.01, and *** indicates P-values less than 0.001. Untreated cells were used as controls. (TIF) [file pone.0232930.s001.tif]

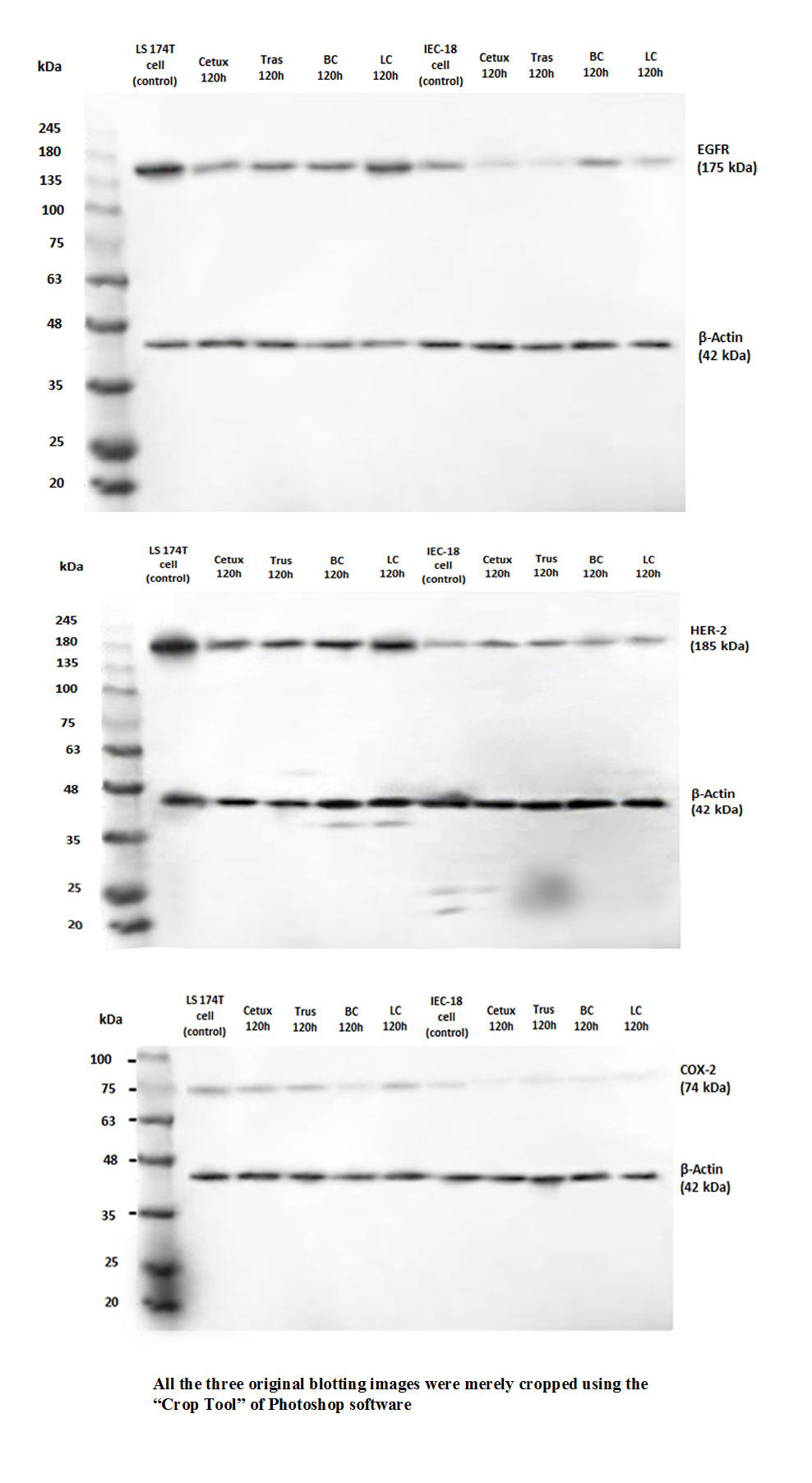

Supplement: S1 Raw images — (TIF) [file pone.0232930.s004.tif]
