# Supplementary material for: Anti-cancer effects of Bifidobacterium species in colon cancer cells and a mouse model of carcinogenesis
Source: PLoS One. 2020 May 13;15(5):e0232930. doi: 10.1371/journal.pone.0232930 (PMC7219778; doi:10.1371/journal.pone.0232930)
Supplement: S2 Fig — Cells considered as viable were Annexin V and PI negative; cells in early apoptosis stage were Annexin V positive and PI negative; and cells in late apoptosis/ necrosis stage were both Annexin V and PI positive. Untreated cells were used as negative controls and cetuximab and trastuzumab were used as positive controls. (PDF) [file pone.0232930.s002.pdf]

S2 Fig.

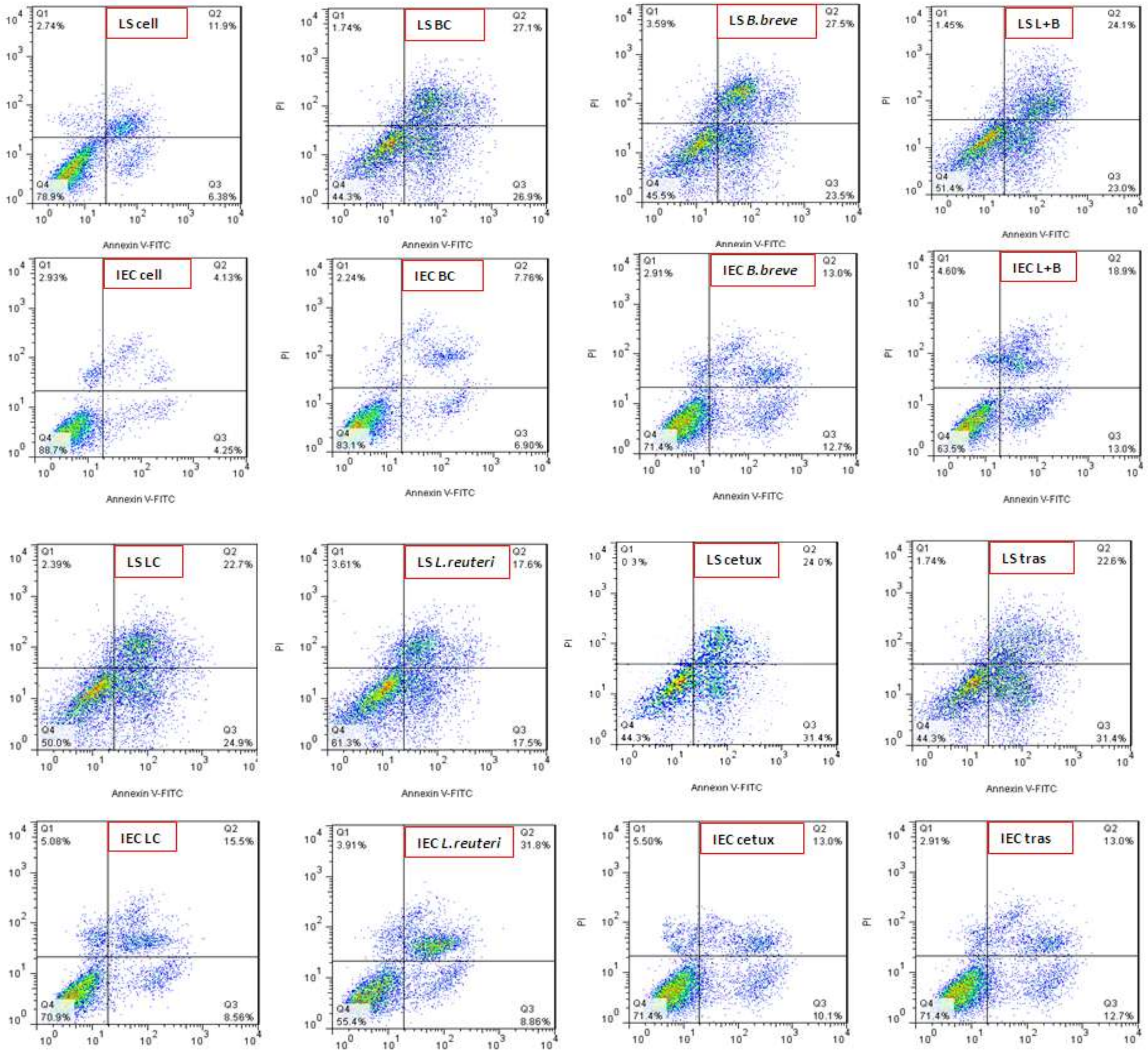

LS: LS174T cancer cells; IEC: IEC-18 normal cells; BC: Bifidobacteria cocktail, LC: Lactobacilli cocktail, L+B: Lactobacilli plus Bifidobacteria cocktail; cetux: cetuximab; tras: trastuzumab
