# Supplementary material for: Anti-cancer effects of Bifidobacterium species in colon cancer cells and a mouse model of carcinogenesis
Source: PLoS One. 2020 May 13;15(5):e0232930. doi: 10.1371/journal.pone.0232930 (PMC7219778; doi:10.1371/journal.pone.0232930)
Supplement: S1 Table — (PDF) [file pone.0232930.s003.pdf]

S1 Table.

	LS174T cells					IEC-18 cells				
	Control	Cetux. 120h	Tras. 120h	BC 120h	LC 120h	Control	Cetux. 120h	Tras. 120h	BC 120h	LC 120h
**EGFR**	1	0.19	0.35	0.4	0.9	1	0.37	0.2	0.007	0.56
**HER-2**	1	0.43	0.50	0.48	0.79	1	5.7	5	1.4	4.9
**COX-2**	1	0.51	0.52	0.37	0.5	1	0.4	0.2	0.7	0.005

Cetux: cetuximab, Tras: trastuzumab, BC: Bifidobacterial cocktail, LC: Lactobacilli cocktail
